# Estetrol/GPER/SERPINB2 transduction signaling inhibits the motility of triple-negative breast cancer cells

**DOI:** 10.1186/s12967-024-05269-6

**Published:** 2024-05-13

**Authors:** Francesca Cirillo, Asia Spinelli, Marianna Talia, Domenica Scordamaglia, Maria Francesca Santolla, Fedora Grande, Bruno Rizzuti, Marcello Maggiolini, Céline Gérard, Rosamaria Lappano

**Affiliations:** 1 Department of Pharmacy, Health and Nutritional Sciences, University of Calabria, Rende, 87036 Italy; 2 Department of Physics, CNR-NANOTEC, SS Rende (CS), University of Calabria, Rende, CS 87036 Italy; 3 Institute of Biocomputation and Physics of Complex Systems (BIFI), University of Zaragoza, Zaragoza, 50018 Spain; 4 Mithra Pharmaceutical, Rue Saint-Georges 5, Liège, 4000 Belgium

**Keywords:** Estetrol (E4), G protein-coupled estrogen receptor (GPER), SERPINB2, Triple-negative breast cancer (TNBC)

## Abstract

**Background:**

Estetrol (E4) is a natural estrogen produced by the fetal liver during pregnancy. Due to its favorable safety profile, E4 was recently approved as estrogenic component of a new combined oral contraceptive. E4 is a selective ligand of estrogen receptor (ER)α and ERβ, but its binding to the G Protein-Coupled Estrogen Receptor (GPER) has not been described to date. Therefore, we aimed to explore E4 action in GPER-positive Triple-Negative Breast Cancer (TNBC) cells.

**Methods:**

The potential interaction between E4 and GPER was investigated by molecular modeling and binding assays. The whole transcriptomic modulation triggered by E4 in TNBC cells via GPER was explored through high-throughput RNA sequencing analyses. Gene and protein expression evaluations as well as migration and invasion assays allowed us to explore the involvement of the GPER-mediated induction of the plasminogen activator inhibitor type 2 (SERPINB2) in the biological responses triggered by E4 in TNBC cells. Furthermore, bioinformatics analysis was aimed at recognizing the biological significance of SERPINB2 in ER-negative breast cancer patients.

**Results:**

After the molecular characterization of the E4 binding capacity to GPER, RNA-seq analysis revealed that the plasminogen activator inhibitor type 2 (SERPINB2) is one of the most up-regulated genes by E4 in a GPER-dependent manner. Worthy, we demonstrated that the GPER-mediated increase of SERPINB2 is engaged in the anti-migratory and anti-invasive effects elicited by E4 in TNBC cells. In accordance with these findings, a correlation between SERPINB2 levels and a good clinical outcome was found in ER-negative breast cancer patients.

**Conclusions:**

Overall, our results provide new insights into the mechanisms through which E4 can halt migratory and invasive features of TNBC cells.

## Background

Estrogens are the main drivers of the development and regulation of female reproduction [1]. In addition, these steroids exhibit pleiotropic effects on non-reproductive tissues including the cardiovascular, neuroendocrine, skeletal, hepatic, and immune systems [1]. Dysregulated estrogen levels are also associated with an increased incidence of diverse tumors including breast cancer [2–4]. The predominant circulating estrogen in the human body is 17β-estradiol (E2), which is the most biologically active estrogen produced by the ovaries during the reproductive years [5]. Estriol (E3) and estetrol (E4) are mainly produced during pregnancy, whereas estrone (E1) increases during menopause [5].

The effects of estrogens are mainly mediated by the nuclear estrogen receptor (ER)α and ERβ, which act as transcription factors toward the regulation of target genes [6]. Aside from the aforementioned signaling, which is referred to as the genomic pathway, estrogens can elicit rapid responses by engaging membrane-initiated signaling [7–9]. In this regard, the G Protein-Coupled Estrogen Receptor (GPER) has recently been enrolled among the mediators of rapid estrogen action in diverse normal and neoplastic cells, including in breast cancer [10–12]. To date, GPER signaling has been shown to trigger intracellular molecular events leading to the transcription of genes implicated in cell proliferation, invasion, metastasis, angiogenesis, and tumor-promoting inflammation [12–17]. In addition, GPER activation has been demonstrated to induce calcium mobilization, cAMP synthesis, the cleavage of matrix metalloproteinases, the transactivation of epidermal growth factor receptor (EGFR), and the activation of the PI3K and MAPK transduction pathways [8, 12]. Estrogens, phyto- and xeno-estrogens can bind and activate GPER [18], albeit E3 may act as a GPER antagonist in breast cancer cells [19].

E4 is a weak natural estrogen produced by the fetal liver during pregnancy [20]. A constant increase of E4 during pregnancy in maternal plasma has been reported and, at term, fetal E4 levels are substantially higher than maternal levels [21]. E4 was approved recently as the estrogenic component of a new combined oral contraceptive and offers an improved safety profile compared to other estrogens. E4 also entered late-stage clinical studies for use as menopausal hormone therapy (MHT) [22, 23].

E4 exhibits a selective binding affinity for ERα and ERβ, with a 4 to 5-fold higher affinity for ERα [24]. The binding affinity of E4 as well as its estrogenic potency are lower compared with E2. In the context of breast cancer, E4 has been described to exhibit mixed agonist and antagonist actions with the capacity to antagonize E2-induced proliferation and migration in ER-positive breast cancer cells both in vitro and in vivo [25, 26]. Moreover, E4 exhibited pro-apoptotic properties in endocrine-resistant breast cancer cells [27, 28]. E4 was also shown to trigger rapid non-genomic effects regardless of ERα, like the activation of the MAPK ERK1/2 and PI3K/AKT pathways in breast cancer cells [25] as well as the GPER-mediated phosphorylation of the focal adhesion kinase (FAK) in endothelial cells [25, 29].

To gain novel insights into this intricate scenario, in the present study we have dissected the transcriptional and biological changes prompted by E4 through GPER in the aggressive triple-negative breast cancer (TNBC) cells. Of note, we have disclosed that GPER mediates the induction of the plasminogen activator inhibitor type 2 (SERPINB2) by E4 toward anti-migratory and anti-invasive effects elicited in TNBC cells. Nicely fitting with these data, bioinformatics analysis from a large cohort of patients assessed that high SERPINB2 levels correlate with an improved outcome in ER-negative breast tumors. Overall, these findings provide novel insights into the potential of E4 to exert through GPER anti-tumor effects in TNBC cells. Nevertheless, further studies are warranted to understand the action of E4 in TNBC cells.

## Methods

### Molecular docking

Molecular docking and all-atom molecular dynamics (MD) simulation were performed by following a previously described protocol for modeling GPER-ligand complexes [30]. In brief, the structure of GPER was modeled using the GPCR-I-TASSER server [31], which is specific for G protein-coupled receptors. The resulting seven-helix transmembrane bundle was considered (residues 50–375), whereas the disordered extracellular N-terminal region (residues 1–49) was neglected. An initial prediction of the binding location of the ligand (E4) was obtained through molecular docking, carried out by using the software AutoDock Vina, version 1.1.2 [32]. .

Up to this point, the molecular system including both GPER and E4 was modeled with apolar hydrogens subsumed into carbon atoms. The compound was placed in the binding pocket open to the extracellular protein region, by performing a search in a volume of 32 Å × 44 Å × 54 Å, at very high (16 times the default value) exhaustiveness in the exploration [33]. Subsequently, apolar hydrogen was added, as well as explicit water and counterions, and the system was relaxed by performing an MD simulation with the GROMACS package [34]. The AMBER ff99SB-ILDN and GAFF force fields were used for the protein and ligand, respectively, and other simulation conditions (including integration of the equation of motions, treatment of electrostatic and van der Waals forces, modeling of long-range London dispersion interactions, and coupling with a barostat and thermostat) were as previously described for other similar protein-ligand complexes [35]. Equilibration of the system was carried out in the isobaric-isothermal ensemble for 5 ns. At the end of the MD simulation, the binding affinity of the ligand was evaluated by re-using the AutoDock Vina scoring function. In parallel to E4, the anchoring mode in the same binding cavity of two reference GPER ligands, i.e. the agonist 17β-estradiol (E2) and the antagonist G15, was assessed.

### Reagents

Estetrol (E4) was provided by Mithra Pharmaceuticals (Liège, Belgium). 17β-Estradiol (E2) was purchased from Merck (Milan, Italy). The GPER agonist G1 (1-[4-(-6-bromobenzol [1, 3] diodo-5-yl)-3a,4,5,9b-tetrahidro3H5cyclopenta[c]quinolin-8yl]-ethanone) and antagonist G15 (3aS,4R,9bR)-4-(6-Bromo-1, 3-benzodioxol-5-yl)-3a,4,5,9b-3 H-cyclopenta[c]quinolone) were purchased from Tocris Bioscience (Merck, Milan, Italy). The MEK inhibitor trametinib was obtained from MedChemExpress (DBA, Milan, Italy). All compounds were dissolved in DMSO, except E2 and E4, which were solubilized in ethanol.

### Cell cultures

MDA-MB-231 and MDA-MB-468 cells were obtained from ATCC (Manassas, VA, USA) and maintained in DMEM/F-12 (Thermo Fisher Scientific, Life Technologies, Milan, Italy), supplemented with 5% fetal bovine serum (FBS) and 100 µg/ml penicillin/streptomycin. Cells were used less than six months after resuscitation and mycoplasma negativity was tested monthly. GPER knockout (KO) MDA-MB-231 cells were generated by employing CRISPR-Cas9 gene-editing system and maintained as previously reported [36]. All cells were grown in a 37 °C incubator with 5% CO_2_ and switched to a medium without serum and phenol red the day before treatments to be processed for immunoblot and RT-PCR assays.

### Ligand binding assay

MDA-MB-231 cells were grown in 10-cm cell culture dishes, washed two times, and incubated with 1 nM [2,4,6,7–3 H]E2 (89 Ci/mmol; Ge Healthcare, Milan, Italy) in the presence or absence of an increasing concentration of unlabeled competitors (E2, G1 and E-4). Then, cells were incubated for two hours at 37 °C and washed three times with ice-cold PBS; the radioactivity collected by 100% ethanol extraction was measured by liquid scintillation counting. Competitor binding was expressed as a percentage of maximal specific binding. Each point is the mean of three observations.

### Gene silencing experiments

Cells were plated into 10-cm dishes, maintained in serum-free medium for 24 h, and then transfected for an additional 48 h before treatments using X-tremeGENE 9 (according to the manufacturer’s recommendations) and control vector (shRNA) or shGPER (Merck, Milan, Italy).

### Gene expression studies

Total RNA was extracted and cDNA was synthesized by reverse transcription as described in our previous work [37]. The expression of selected genes was quantified by real-time PCR using the platform Quant Studio7 Flex Real-Time PCR System (Thermo Fisher Scientific, Milan, Italy). Gene-specific primers were designed using Primer Express version 2.0 software (Applied Biosystems) and are as follows: 5′-AAGCCACCCCACTTCTCTCTAA-3′ (ACTB forward) and 5′-CACCTCCCCTGTGTGGACTT-3′ (ACTB reverse); 5′-ATGAGGAGGGCACTGAAGCA-3′ (SERPINB2 forward) and 5′-TGTCCAGTTCTCCTGTCATACA-3′ (SERPINB2 reverse). Assays were performed in triplicate and the results were normalized for actin beta (ACTB) expression and then calculated as fold induction of RNA expression.

### RNA-seq pipeline

Total RNA from MDA-MB-231 cells was extracted using a RNeasy mini kit according to the manufacturer’s instructions (Qiagen, Bioset s.r.l., Catanzaro, Italy). RNA integrity for library preparation was determined by analysis of extracted total RNA using a 2100 Bioanalyzer (Agilent Technologies) with RNA 6000 NanoChip. RNA concentrations were measured using the Qubit RNA Assay Kit. Libraries were prepared from total RNA according to manufacturer instructions with Illumina Stranded mRNA Prep kit. Libraries quality was evaluated by size analysis on 2100 Bioanalyzer (Chip DNA HS) and concentrations were determined using a Qubit DNA HS assay kit (Thermo Fisher Scientific). Sequencing was performed on Illumina Novaseq 6000 in the 100PE format. Reads preprocessing was performed by using fastp v0.20.0 [38], applying specific parameters to remove residual adapter sequences and to keep only high-quality data (qualified_quality_phred = 20, unqualified_percent_limit = 30, average_qual = 25, low_complexity_filter = True, complexity_threshold = 30). The percentage of uniquely mapped reads resulted high with mean value of 84% (mean value for sample: 60 million total reads, unmapped reads 7%, quality base > q30 94%). Then, passing filter reads were mapped to the human genome reference (version GRCh38) using STAR v2.7.0 [39] with standard parameters, except for sjdbOverhang option set on read length. Genome and transcripts annotation provided as input were downloaded from v99 of Ensembl repository. Alignments were then elaborated by RSEM v1.3.3 [40] to estimate transcript abundances. Subsequently, the sample-specific gene-level abundances were merged into a single raw expression matrix by applying a dedicated RSEM command (rsem-generate-data-matrix). Genes with at least 10 counts in 50% of samples were then selected. Differential expression (pairwise comparisons) was computed by edgeR [41] from raw counts in each comparison. Re-annotation of previously differentially expressed genes (DEGs) was performed using the bioMart package [42] into R 3.6, querying available Ensembl Gene IDs and retrieving Gene Names and Entrez gene IDs.

### Data collection

Data from the TCGA and Affymetrix datasets were used in the current study. mRNA expression data (RNA Seq V2 RSEM) and associated clinical information reported in the Invasive Breast Cancer Cohort of the TCGA project were retrieved from UCSC Xena (https://xenabrowser.net/) on the 19th of July 2023. The gene expression levels and clinical information of the Affymetrix cohort were retrieved from 17 integrated Affymetrix gene expression datasets, as previously described [43]. In brief, the Raw.cel files from 17 Affymetrix U133A/plus two gene expression datasets of primary breast tumors were retrieved from NCBI GEO (GSE12276, GSE21653, GSE3744, GSE5460, GSE2109, GSE1561, GSE17907, GSE2990, GSE7390, GSE11121, GSE16716, GSE2034, GSE1456, GSE6532, GSE3494), summarized with Ensembl alternative CDF, and then normalized with RMA, before their integration using ComBat to eliminate dataset-specific bias [43]. Breast cancer patients of both TCGA and Affymetrix were classified based on the presence or the absence of the estrogen receptor (ER). Gene expression and clinical information were filtered for missing values. TCGA samples were also filtered by “sample type” in order to separate tumor tissues from the adjacent normal tissues. All of the bioinformatics analyses were carried out using R Studio (version 4.1.3).

### Survival analysis

The survival analysis was performed using the SERPINB2 gene expression levels of the Affymetrix ER-negative breast cancer patients along with the distant metastasis-free survival (DMFS) data. The survival analysis on SERPINB2 expression has been performed using the survivALL package bioRxiv 208,660 through which Cox proportional hazards for all possible points-of‐separation (low‐high cut‐points) were examined. Therefore, samples with high (*n* = 90) and low (*n* = 80) SERPINB2 expression levels were separated according to the most significant cut-point. The Kaplan Meier survival curves were generated using the survival and the survminer R packages.

### Pathway enrichment analysis

To investigate the biological significance of the differentially expressed genes (DEGs) arising from the RNA-seq analysis, the Reactome package [44] was employed in R Studio to assess the signaling pathway enrichment analysis. The following parameters were used: organism = “human”, p-value cut-off = 0.05, q-value cut-off = 0.2.

### Western blotting analysis

Cells were grown in 10-cm dishes, exposed to treatments where required, and then lysed in 500 µl RIPA buffer with protease inhibitors (1.7 mg/ml aprotinin, 1 mg/ml leupeptin, 200 mmol/l phenylmethylsulfonyl fluoride, 200 mmol/l sodium orthovanadate, and 100 mmol/l sodium fluoride). Samples were then centrifuged at 13,000 rpm for 10 min and protein concentrations were determined using BCA protein assay according to the manufacturer’s instructions (Thermo Fisher Scientific, Milan, Italy). Equal amounts of whole-protein extract were resolved on 10% SDS polyacrylamide gels and transferred to nitrocellulose membranes (Merck Life Science, Milan, Italy), which were probed with primary antibodies against SERPINB2 (ab47742), GPER (ab137479) (Abcam, DBA, Milan, Italy), p-ERK1/2 (E-4), ERK2 (C-14) and β-actin (AC-15) (Santa Cruz Biotechnology, DBA, Milan, Italy). Proteins were detected by horseradish peroxidase-linked secondary antibodies (Bio-Rad, Milan, Italy) and then revealed using the chemiluminescent substrate for western blotting Clarity Western ECL Substrate (Bio-Rad, Milan, Italy). Densitometric analysis was performed using the freeware software ImageJ that allowed the quantification of the band intensity of the protein of interest with respect to the band intensity of the loading control.

### Phalloidin staining

Cells were exposed to treatments for 15 h washed twice with PBS, fixed in 4% paraformaldehyde in PBS for 10 min, washed briefly with PBS, then incubated with Phalloidin-Fluorescent Conjugate (Santa Cruz Biotechnology, DBA, Milan, Italy). The images were obtained using the Cytation 3 Cell Imaging Multimode reader (BioTek, AHSI, Milan Italy) and analyzed by the Gen5 software (BioTek, AHSI, Milan Italy).

### Migration and invasion assays

Transwell 8 μm polycarbonate membranes (Costar, Merck Life Science) were used to evaluate in vitro cell migration and invasion. 5 × 10^4^ cells in 300 µL serum-free medium were seeded in the upper chamber coated with (invasion assay) or without (migration assay) Corning® Matrigel® Growth Factor Reduced (GFR) Basement Membrane Matrix (Merck Life Science) (diluted with a serum-free medium at a ratio of 1:3). Medium containing 2.5% FBS and treatments was added to the bottom chambers. 6 h after seeding, cells on the upper surface of the membrane were then removed by wiping with Q-tip, and migrated or invaded cells were fixed with 100% methanol, stained with Giemsa (Merck Life Science), photographed using an inverted phase contrast microscope and counted using the WCIF ImageJ software.

### Statistical analysis

Statistical analyses were performed using t-tests or ANOVA followed by Newman-Keuls’ test to determine differences in means. The heatmap was drawn with the pheatmap R package, bar plots, and volcano plot were performed with the R tidyverse package. Box plots were performed with the R tidyverse package and the related statistical analyses were calculated using the Wilcoxon test. p-values < 0.05 were considered statistically significant.

## Results

### Molecular modeling and binding assays suggest that E4 binds to GPER

**Fig. 1:**
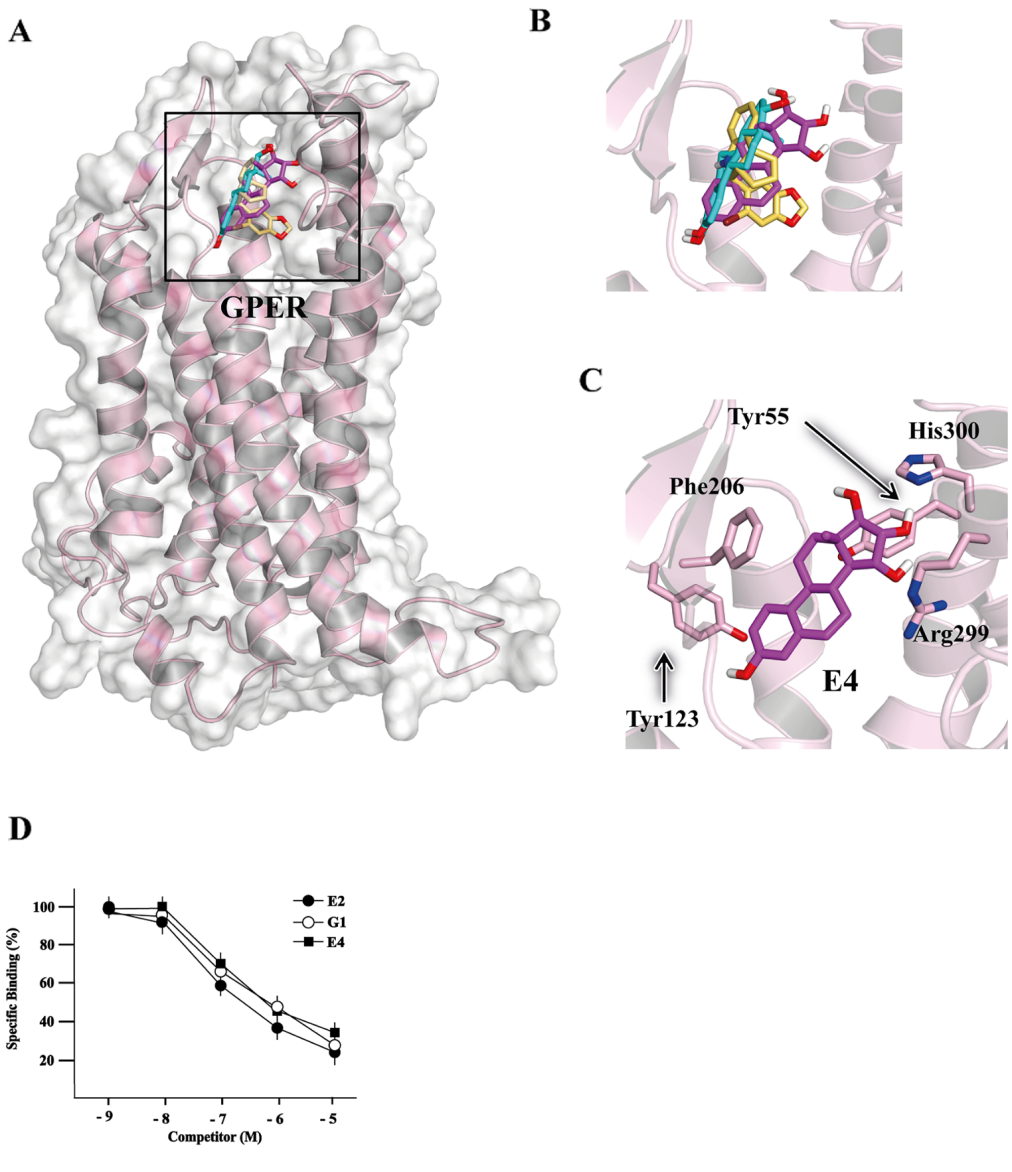
E4 acts as a ligand of GPER. (**A**) Superimposed binding modes of E4 (magenta), E2 (cyan) and G15 (yellow) in the GPER binding site. The sole seven-helix transmembrane structure of GPER (residues 50–375) is shown. (**B**) Magnification of the conformations of the three ligands in the binding pocket. (**C**) Detail of the binding mode of E4, with the interacting protein residues evidenced. (**D**) Ligand binding assay in MDA-MB-231 cells. Competition curves at increasing concentrations of E2, G1 and E4 expressed as a percentage of maximum specific [3 H] E2 binding. Each point represents the mean ± SD of three separate experiments performed in triplicate

Considering that E4 has gained attention as a potential therapeutic agent in diverse conditions, including contraception and menopause, useful evidence may be provided dissecting its action and biological effects mediated by GPER. Therefore, we began our study by performing molecular docking and molecular dynamics (MD) simulations as well as ligand binding assays to evaluate the ability of E4 to bind to GPER. We used a 3-dimensional model of GPER that we have previously modeled to investigate the binding mode of this protein to several ligands [30, 45]. Molecular docking was carried out on a selected region of GPER, which includes the whole binding pocket common to any of its ligands [33]. The simulation led to an anchoring mode of E4 with a binding affinity of ‒7.0 kcal/mol. Such a starting prediction was later refined by performing an MD simulation of the protein-ligand complex for 5 ns. In this way, E4 was left free to accommodate within the binding pocket of GPER, taking also into account the dynamics of the whole protein. At the end of the MD simulation, the binding affinity of the ligand in the anchoring geometry obtained was re-evaluated. The docking in the same binding pocket of two ligands of GPER, the agonist E2 and the antagonist G15, was evaluated as well. The results shown in Fig. 1 (panels A-B) indicate that E4, E2, and G15 are all able to accommodate in the same binding site, and with evident similarities in their binding modes. In particular, E4 interacts with key residues of GPER that were previously identified to be involved in the association of other ligands. The interactions that mainly contributed to the binding energy include the formation of a hydrogen bond established with the side chain of protein residue His300 as acceptor, as well as hydrophobic interactions formed with the side chain of Tyr55. Other charged (e.g., Arg299) and aromatic residues (e.g., Tyr123 and Phe206) also participate in the binding (Fig. 1C). The binding energy of E4 in this position, evaluated with a redocking in score-only mode [32], was ‒6.9 kcal/mol. This value is almost identical to the one previously found with the starting docking procedure, although the two situations differ because during the MD simulation, both the ligand and the interacting protein residues may not rest in an ideal geometry, due to their dynamics. The uncertainty on the calculated binding affinity, estimated by sampling different structures of the protein-ligand complex in the last 100 ps of simulation, was found to be in the order of 0.5 kcal/mol.

Under similar conditions, the binding energies calculated for E2 and G15 were ‒7.5 and ‒7.2 kcal/mol, respectively, which are values slightly more favorable than the one found for E4 if the uncertainty in the docking score algorithm (~ 0.3 kcal/mol) is considered, and roughly within the same range if thermal agitation effects at room temperature (~ 0.6 kcal/mol) are taken also into account. It is important to note that all these values underestimate the experimental binding affinity, which is expected to be in the order of a nanomolar dissociation constant for E2 [46]. In conclusion, E4 shows a behavior towards the target protein not only similar to E2 and G15, but also to that previously observed for other known GPER ligands subjected both to computational modeling and to validation of the results through in vitro studies [33]. Overall, these findings support the ability of E4 to interact with GPER. To further corroborate these data by competitive binding assays, we used as a model system MDA-MB-231 cells that endogenously express GPER but lack ERα and ERβ, as previously reported [36, 47]. E4 displaced radiolabeled E2, which was used as a tracer, with quite similar affinities with respect to E2 and the GPER agonist G1 (Fig. 1D). Cumulatively, the aforementioned results provide comprehensive findings on the ability of E4 to bind to GPER.

### Transcriptome analysis discloses that E4 up-regulates SERPINB2 expression through GPER in TNBC cells

**Fig. 2:**
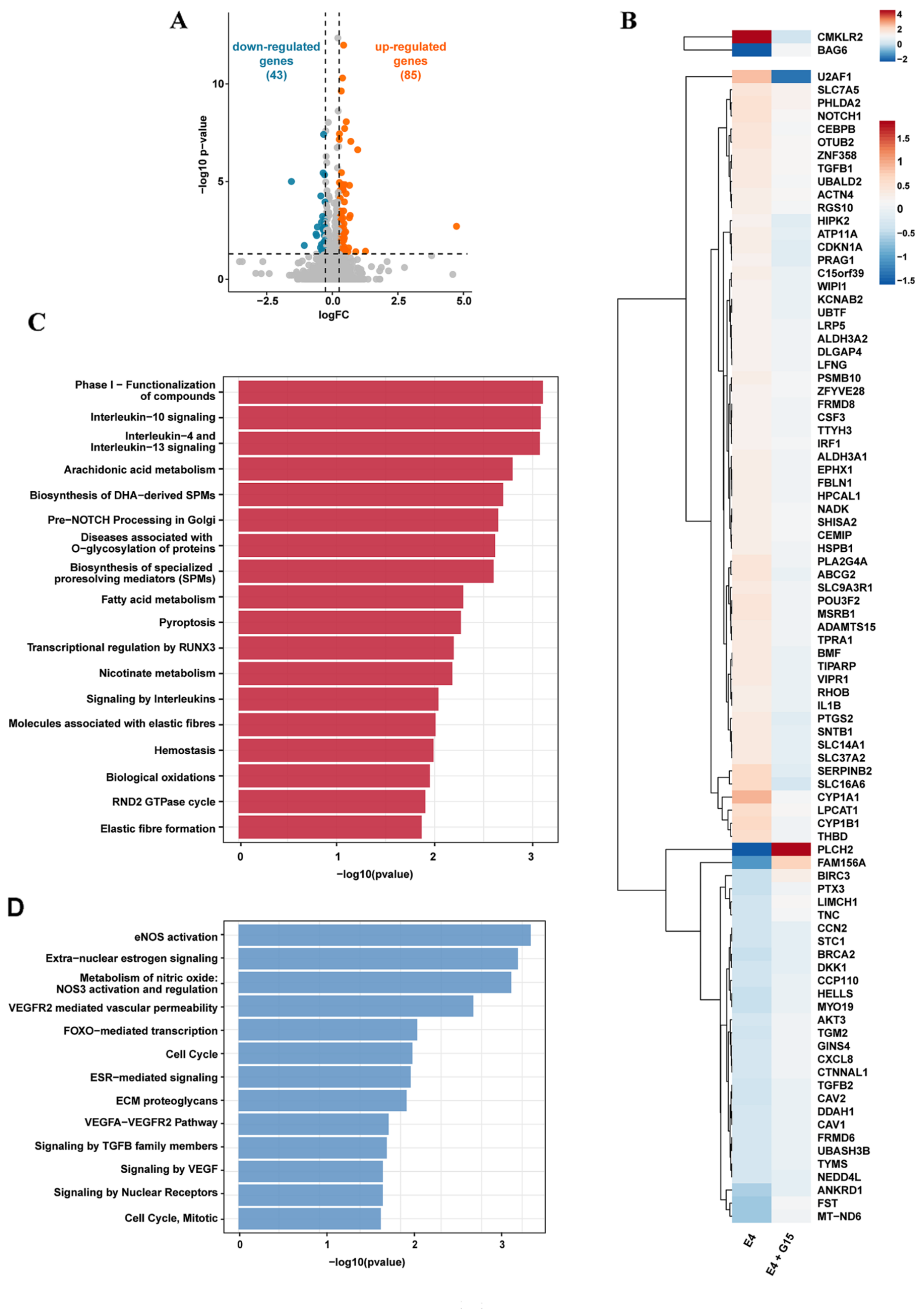
RNA-seq based transcriptomic profiling of MDA-MB-231 cells exposed to E4 through GPER//RNA-seq based transcriptomic profiling of E4-treated MDA-MB-231 cells via GPER. (**A**) Volcano plot showing the differentially expressed genes (DEGs) in MDA-MB-231 cells treated for 8 h with 100 nM of E4, as assessed by RNA-seq analysis. (**B**) Heat map depicting genes up-regulated by E4 and inhibited in the presence of 100 nM of G15 (*n* = 60) as well as genes down-regulated by E4 and rescued in the presence of G15 (*n* = 30). (**C**) Clustering of the genes induced by E4 and inhibited by G15 in MDA-MB-231 cells, as ascertained by Reactome pathway analysis. (**D**) Clustering of genes down-regulated by E4 and rescued in the presence of G15 in MDA-MB-231 cells, as evaluated by Reactome pathway analysis. The − log10 p-values are displayed on x-axis, while the different Reactome pathways are shown on y‐axis. *p* < 0.05 was considered statistically significant

**Fig. 3:**
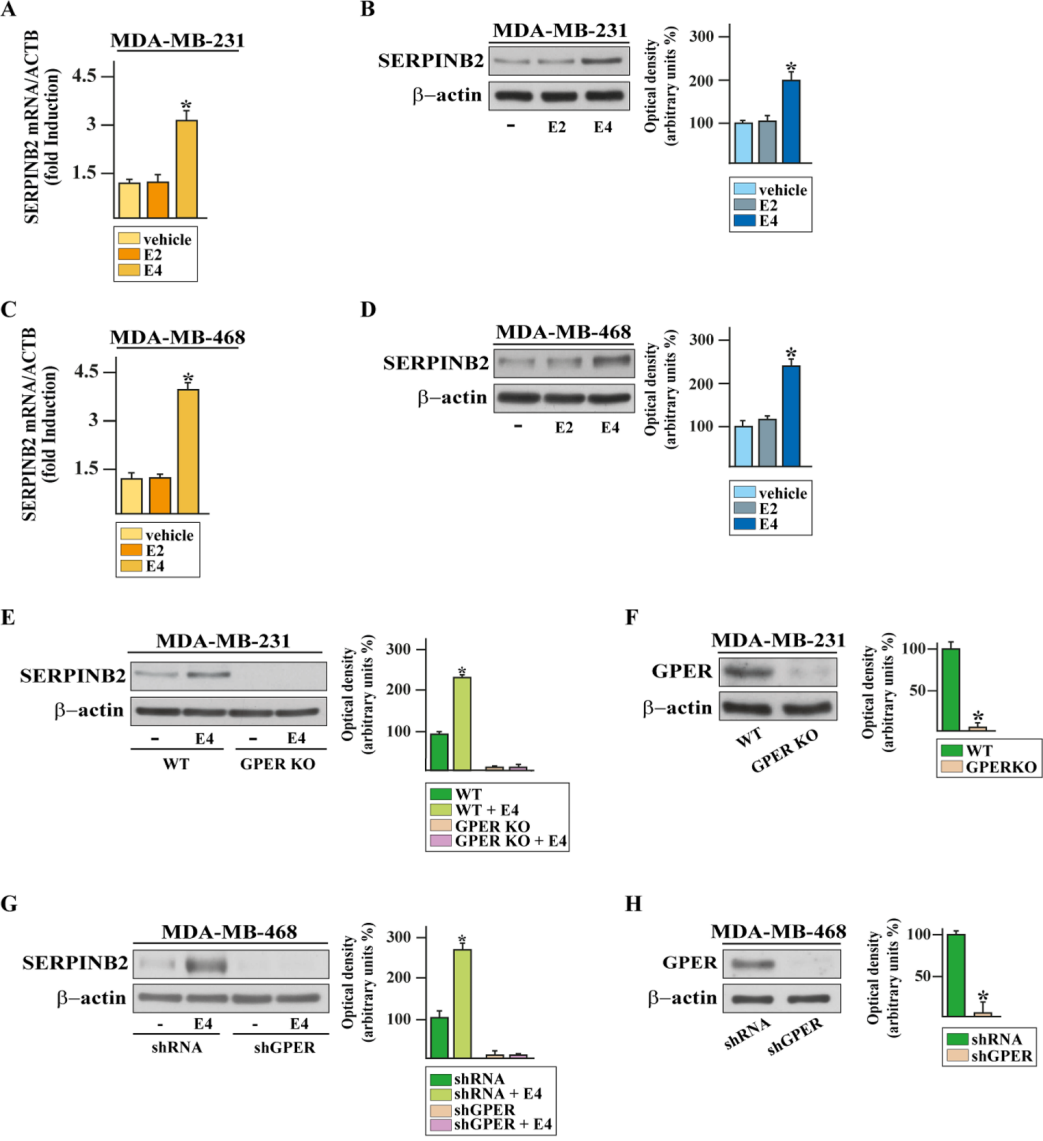
GPER is involved in the up-regulation of SERPINB2 by E4 in TNBC cells. mRNA (**A**, **C**) and protein (**B**,**D**) expression of SERPINB2 in MDA-MB-231 (**A**-**B**) and MDA-MB-468 (**C**-**D**) treated for 8 h with vehicle (-) or 100 nM of E2 and E4, as ascertained by real-time PCR and immunoblotting experiments, respectively. In RNA experiments, values are normalized to the actin beta (ACTB) expression and shown as fold changes of SERPINB2 mRNA expression upon E2 and E4 compared to vehicle-exposed cells. (**E**) Immunoblot of SERPINB2 from wild type (WT) and GPER knockout (KO) MDA-MB-231 cells treated for 8 h with vehicle (-) or 100 nM of E4. (**F**) Efficacy of GPER silencing in GPER KO MDA-MB-231 cells, which were generated by CRISPR/Cas9-mediated genome editing. (**G**) SERPINB2 protein levels in MDA-MB-468 cells transfected with shGPER for 36 h and then exposed to 8 h with vehicle (-) or 100 nM of E4. (**H**) Efficacy of GPER silencing in MDA-MB-468. Side panels show densitometric analysis of the blots normalized to β-actin, which served as a loading control. Values represent the mean ± SD of three independent experiments. (*) indicates *p* < 0.05

The aforementioned data prompted us to further investigate the biological responses elicited by E4 through GPER in the context of breast cancer. We characterized the yet unexplored transcriptomic profile triggered by E4 through GPER in TNBC cells. In this vein, we performed RNA sequencing (RNA-seq) analysis in MDA-MB-231 cells treated with E4 in the presence or absence of the GPER antagonist G15. Figure 2 (panel A) shows the volcano plot of the differentially expressed genes (DEGs) in MDA-MB-231 cells treated with E4. In particular, 85 genes were found up-regulated (log2FC ≥ 0.26, *p* < 0.05) and 43 genes were found down-regulated (log2FC ≤ -0.26, *p* < 0.05) in MDA-MB-231 cells exposed to E4 compared to vehicle-treated cells. As it concerns the gene expression changes mediated by GPER, we found that G15 inhibits the expression of 60 DEGs up-regulated by E4 and rescues the expression of 30 DEGs inhibited by E4, as shown in the heatmap of Fig. 2 (panel B). To assess the biological significance of the aforementioned genes regulated by E4 through GPER, we performed Reactome pathway analysis that revealed the pathway patterns shown in Fig. 2 (panels C-D). Then, we focused on the plasminogen activator inhibitor type 2 (SERPINB2), which is one of most induced genes by E4 in MDA-MB-231 cells (Fig. 2B). Of note, SERPINB2 has been shown to contribute to diverse physiological processes like blood coagulation, fibrinolysis, inflammation, complement activation, cell survival, differentiation, motility and adhesion [48, 49]. Initially, we aimed to further ascertain the induction of SERPINB2 by E4 at both mRNA (Fig. 3A) and protein (Fig. 3B) levels in MDA-MB-231 cells. Nicely fitting with these data, similar responses were also observed in MDA-MB-468 TNBC cells (Fig. 3C-D). Contrary to E4, E2 was not able to regulate SERPINB2 expression in both MDA-MB-231 (Fig. 3A-B) and MDA-MB-468 (Fig. 3C-D) cells.

To corroborate the involvement of GPER in the up-regulation of SERPINB2 by E4, we turned to GPER KO MDA-MB-231 cells that we generated through the CRISPR/Cas9 genome editing technology [36]. A matched cell line harboring the empty vector (WT cells) was also generated and used as a control. Worthy, E4 was no longer able to stimulate SERPINB2 protein expression in GPER KO MDA-MB-231 cells, contrary to MDA-MB-231 WT cells (Fig. 3E-F). In line with these results, the up-regulation of SERPINB2 by E4 was abolished silencing the expression of GPER in MDA-MB-468 cells (Fig. 3G-H).

**Fig. 4:**
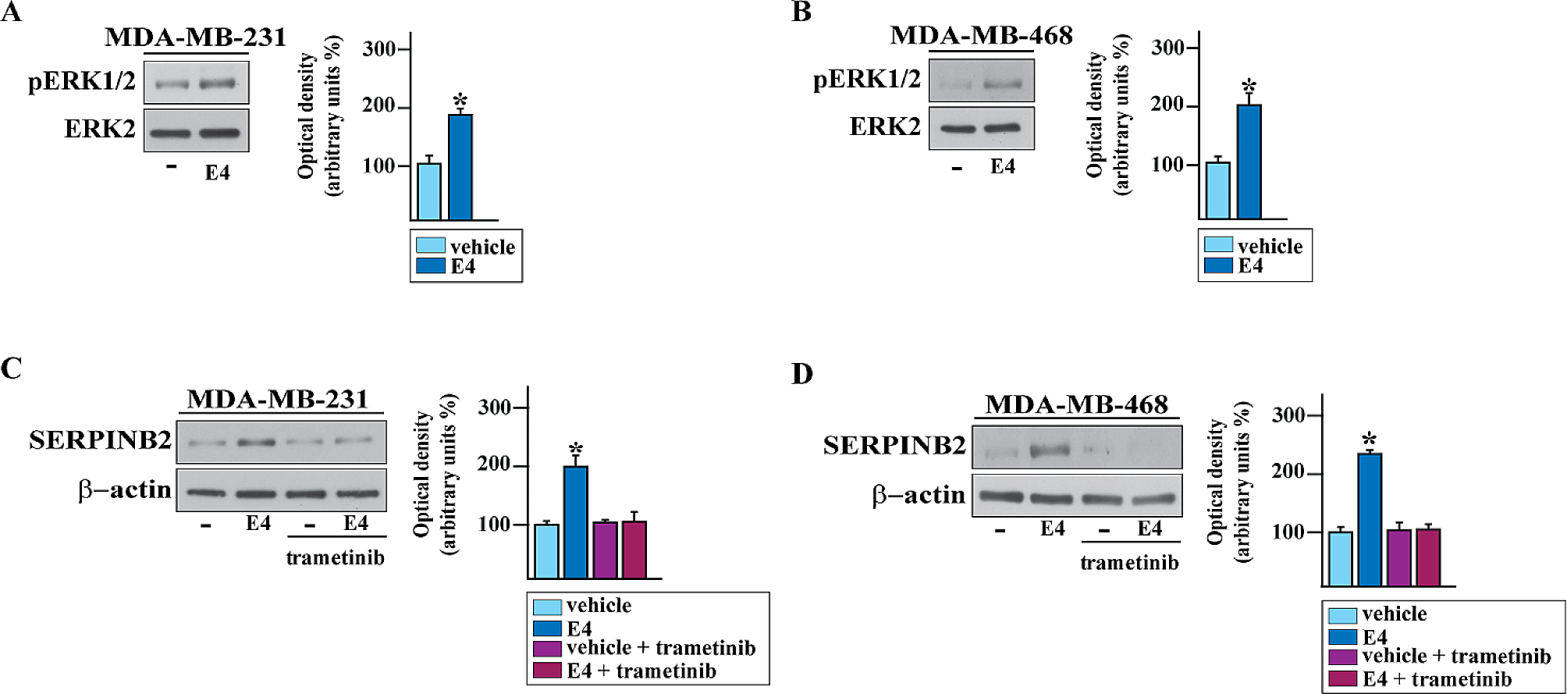
ERK1/2 signaling is required for the E4-induced increase of SERPINB2 in TNBC cells. ERK1/2 phosphorylation in MDA-MB-231 (**A**) and MDA-MB-468 (**B**) cells induced upon exposure to 100 nM of E4 for 20 min. Immunoblots showing SERPINB2 protein expression in MDA-MB-231 (**C**) and MDA-MB-468 (**D**) cells treated for 8 h with vehicle (–) or 100 nM of E4, either alone or in the presence of 100 nM of the MEK inhibitor trametinib. Side panels show densitometric analysis of the blots normalized to the loading controls, as indicated. Values represent the mean ± SD of three independent experiments. (*) indicates *p* < 0.05

Aiming to assess the transduction pathway involved in the induction of SERPINB2 by E4, we determined that E4 triggers ERK activation in both MDA-MB-231 (Fig. 4A) and MDA-MB-468 (Fig. 4B) cells. Next, we demonstrated that the MEK inhibitor trametinib inhibits the SERPINB2 protein increase by E4 in both TNBC cell lines used (Fig. 4C-D), suggesting that E4 up-regulates SERPINB2 expression through the GPER/ERK signaling in TNBC cells.

### SERPINB2 is involved in the inhibitory effects elicited by E4 on the TNBC cell motility

**Fig. 5:**
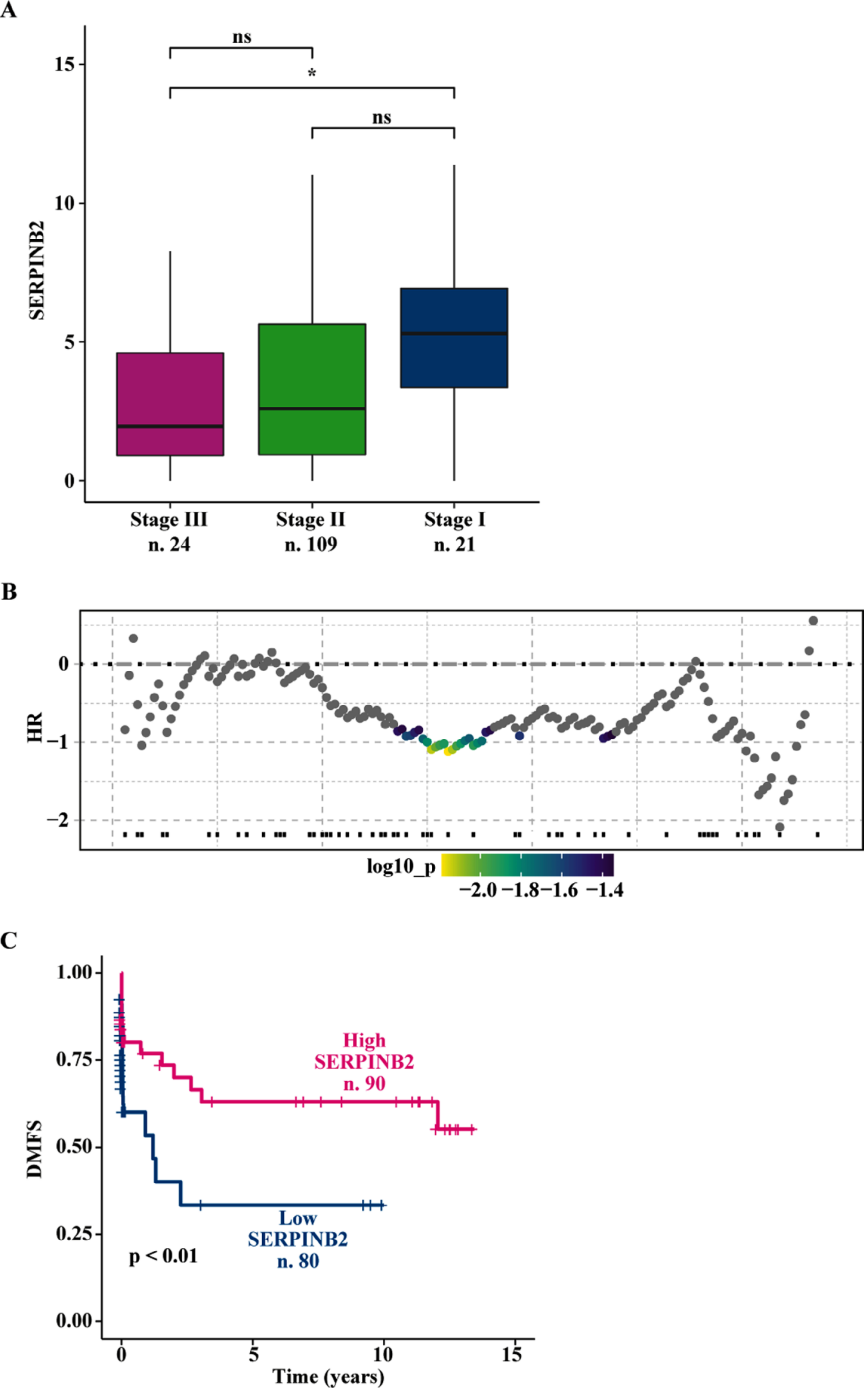
SERPINB2 expression correlates with favorable clinical features in ER-negative breast cancer patients. (**A**) Boxplot depicting the mRNA levels of SERPINB2 in ER-negative breast cancer patients of the TCGA cohort according to tumor stage. p-values are shown in the panels. (**B**) PlotALL function calculating hazard ratio (HR) for all the possible SERPINB2 cut-point to be examined in ER-negative breast cancer patients of the Affymetrix dataset. The color bar gradient stands for the range of the most significant points-of‐separation of the population (low‐high significance = blue‐yellow gradient) based on SERPINB2 expression and distant metastasis-free survival (DMFS) of each patient. The x‐axis represents the patients ordered by the increasing expression of SERPINB2. (**C**) Kaplan-Meier curves showing the correlation between SERPINB2 expression and DMFS in Affymetrix ER-negative breast tumor patients. (*) indicates *p* < 0.05

**Fig. 6:**
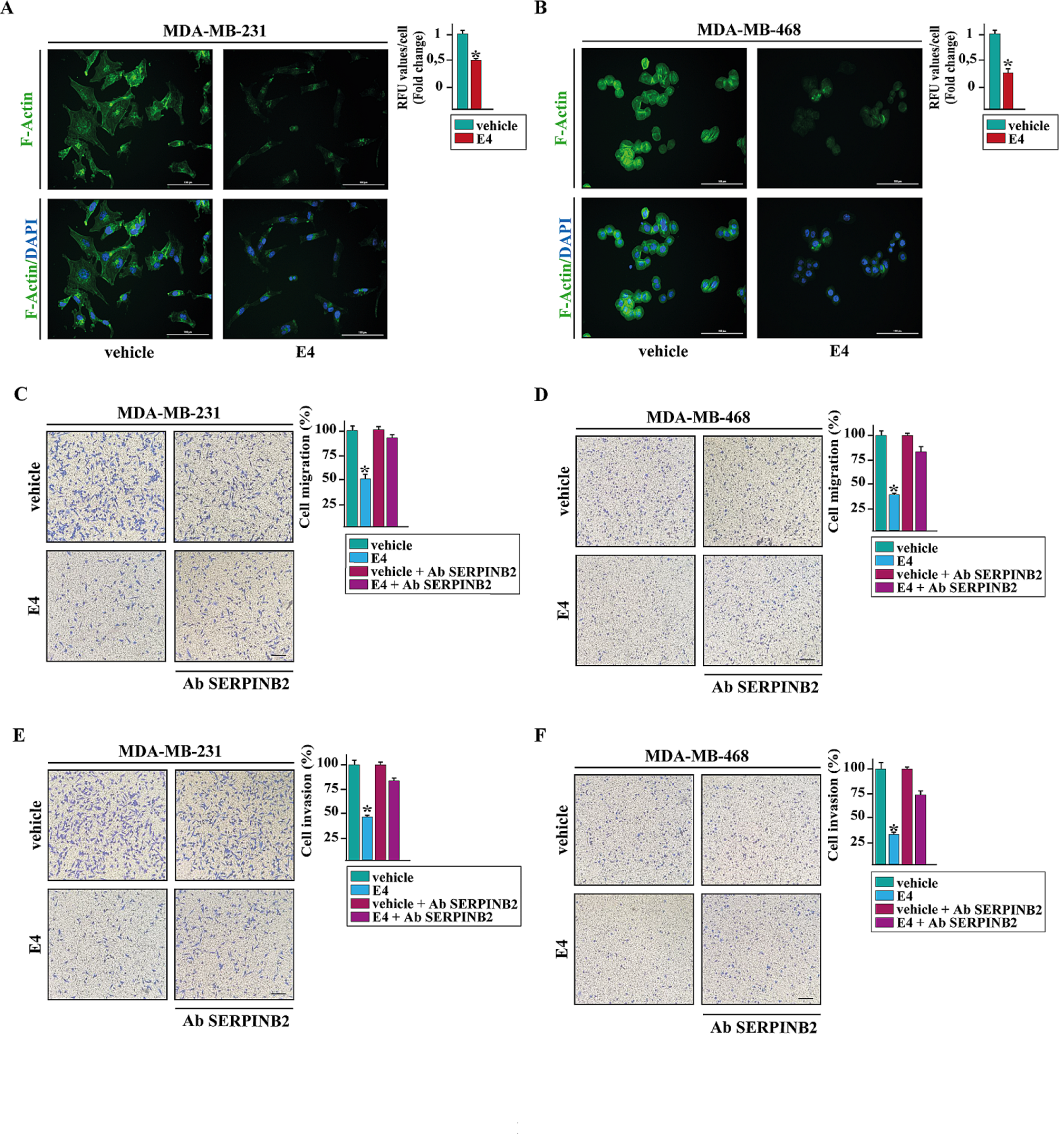
E4 inhibits TNBC cell motility through SERPINB2 induction. MDA-MB-231 (**A**) and MDA-MB-468 (**B**) cells, treated for 24 h with vehicle or 100 nM of E4, were stained with FITC-phalloidin to detect F-actin stress fibers (green) and DAPI to detect nuclei (blue). The number of stress fibers/cell was quantified based on F-actin staining in 20 random fields for each condition; results are expressed as fold change of relative fluorescence units (RFU) (side panel). Data shown represent the mean ± SD of three independent experiments performed in triplicate. Scale bar: 100 µM. Transwell migration (**C**-**D**) and invasion (**E**-**F**) assays in MDA-MB-231 and MDA-MB-468 cells treated for 6 h with vehicle or 100 nM E4, either alone or in combination with 100 ng/mL SERPINB2 antibody. Cells were counted in at least 10 random fields in three independent experiments performed in triplicate, as quantified in the side panels. Scale bar: 200 μm. (*) indicates *p* < 0.05

To appreciate the clinical significance of SERPINB2 in breast cancer, we performed comprehensive bioinformatics analyses on a large cohort of breast tumor patients. In ER-negative breast tumors, we found that high SERPINB2 levels are associated with a low tumor stage (Fig. 5A) and an improved distant metastasis-free survival (DMFS) (Fig. 5B-C). Overall, these findings suggest that SERPINB2 expression may contribute to halting the development of ER-negative breast cancer. Next, we wondered what the biological significance of SERPINB2 increase by E4 might be. On the basis of previous data showing that SERPINB2 can impair cancer cell migration, invasion, and metastasis [50–52], we investigated whether E4 was able to regulate the motile phenotype of TNBC cells through SERPINB2 induction. In this regard, we first investigated the potential of E4 to modify filamentous actin (F-actin), which has been reported to influence cancer cell motility [53]. Of note, a reduction in F-actin was observed MDA-MB-231 (Fig. 6A) and MDA-MB-468 (Fig. 6B) cells exposed to E4. Consistent with these findings, E4 reduced the migration (Fig. 6C, E) and invasion (Fig. 6D, F) of these TNBC cells. To determine whether SERPINB2 might be involved in the aforementioned effects, we used an anti-SERPINB2 antibody (Ab SERPINB2) that rescued the inhibitory action of E4 on cell migration (Fig. 6C, E) and invasion (Fig. 6D, F). Taken together, these data indicate that the GPER/SERPINB2 axis may be considered as a novel mediator of the inhibitory action elicited by E4 on the motility of TNBC cells.

## Discussion

Estrogens are mainly involved in female reproductive functions; however, they also contribute to regulating skeletal maturation and homeostasis, lipid and carbohydrate metabolism, cardiovascular, immune and central nervous systems activities, electrolyte balance and skin integrity [54–56]. Estrogen levels drop during menopause leading to uncomfortable symptoms and increased risk of heart disease, osteoporosis, and fractures [57]. In this context, numerous studies have demonstrated the beneficial effects of estrogen-based hormone replacement therapy (HRT) in the management of menopause-related symptoms [54, 57, 58]. However, randomized trials of the Women’s Health Initiative (WHI) have uncovered the potential risks associated with estrogen-based HRT as well as with contraceptives, which have been both associated with an increased probability of stroke, pulmonary embolism, gallbladder disease, and urinary incontinence [59–62]. In addition, it should be mentioned that an augmented risk of invasive breast cancer has been observed in women who undergo menopausal HRT or utilize estrogen-containing contraceptives [60, 63]. In this intricate scenario, the Food and Drug Administration (FDA) has recently approved the use of E4 in combination with drospirenone (DRSP) as a new combined oral contraceptive due to its efficacy, safety, and user tolerability [64]. E4 is also under development for use as HRT.

As it concerns breast cancer, E4 was shown to exhibit dual agonist/antagonist effects and a specific profile of ERα activation [26, 65]. In this regard, previous studies have shown that E4 exhibits antagonistic properties toward the growth and migration prompted by E2 in ER-positive breast cancer cells [25, 26], whereas it can stimulate a proliferative response in ER-positive breast cancer cells and tumors at concentration doses exceeding those used for therapeutic purposes in menopausal women [25, 66]. In agreement with these findings, E4 has demonstrated anti-tumor effects, along with a safe profile, in postmenopausal patients with advanced and/or metastatic breast tumors resistant to anti-estrogen treatments [67].

Based on the aforementioned data, the present study aimed to focus on a novel route through which E4 may trigger a relevant action in breast cancer. In particular, the potential involvement of the membrane estrogen receptor GPER in mediating the effects of E4 in TNBC cells was disclosed. In this regard, the compelling evidence to explore the role of E4/GPER axis arise from: (i) the established capacity of this receptor to mediate estrogenic signals in both normal and tumor cells, including breast cancer [12]; (ii) the opposite functions that antiestrogens and certain estrogens (i.e. E3) can elicit through ERα and GPER [13, 18, 19]; (iii) the intriguing question concerning the role played by E4 in ER-negative breast cancer such as TNBC cells. Of note, molecular docking simulations and binding assays allowed us to establish the ability of E4 to bind to GPER. Thereafter, genome-wide RNA-seq studies assessed the GPER-mediated transcriptomic landscape of TNBC cells exposed to E4. Interestingly, we also ascertained that E4 stimulates the expression of SERPINB2 in TNBC cells through the GPER/ERK transduction pathway.

The urokinase plasminogen activator (uPA) system comprises the serine protease uPA, its inhibitors PAI-1 (plasminogen activator inhibitor type-1, SERPINE1) and PAI-2 (plasminogen activator inhibitor type-2, SERPINB2) as well as the receptor known as uPAR [68]. This system has been widely involved in the invasion and metastatic spread of cancer cells as well as in tumor cell proliferation, adhesion, and neo-angiogenesis [69, 70]. In this vein, it should be mentioned that uPA is implicated in the conversion of inactive plasminogen to active plasmin, which is a serine protease triggering the degradation of the extracellular matrix (ECM) and therefore allowing the malignant cells to locally invade and metastasize [71].

SERPINE1 and SERPINB2 negatively regulate the uPA/uPAR pathway acting as uPA naturally occurring inhibitors. By binding to uPA, they block the conversion of plasminogen into plasmin, thus playing a crucial role in regulating the balance between clot formation and dissolution, tissue remodeling, and cell migration [72, 73]. SERPINB2 shows a higher affinity constant for uPA with respect to SERPINE1 and appears to be a true protease inhibitor [74]. Numerous experimental evidence demonstrated that high levels of SERPINB2 may inhibit the stimulatory effects induced by uPA in diverse types of tumors [50–52, 75–77]. Clinical observations nicely corroborate these aforementioned data by indicating that high SERPINB2 levels are associated with extended survival, reduced metastasis, and the inhibition of tumor growth, contrary to SERPINE1 that has been correlated with a poor prognosis and metastatic progression [51, 69, 78–81]. Worthy, SERPINB2 expression has been significantly associated with prolonged survival, decreased metastasis, or decreased tumor size in patients with breast cancer [69, 74, 78, 80]. In line with these discoveries, we have found that high levels of SERPINB2 are associated with a low tumor stage and an improved distant metastasis-free survival (DMFS) in ER-negative breast tumors. Reinforcing these data, we have demonstrated that E4 through GPER up-regulates SERPINB2 leading to anti-migratory and anti-invasive responses in TNBC cells.

## Conclusions

**Fig. 7:**
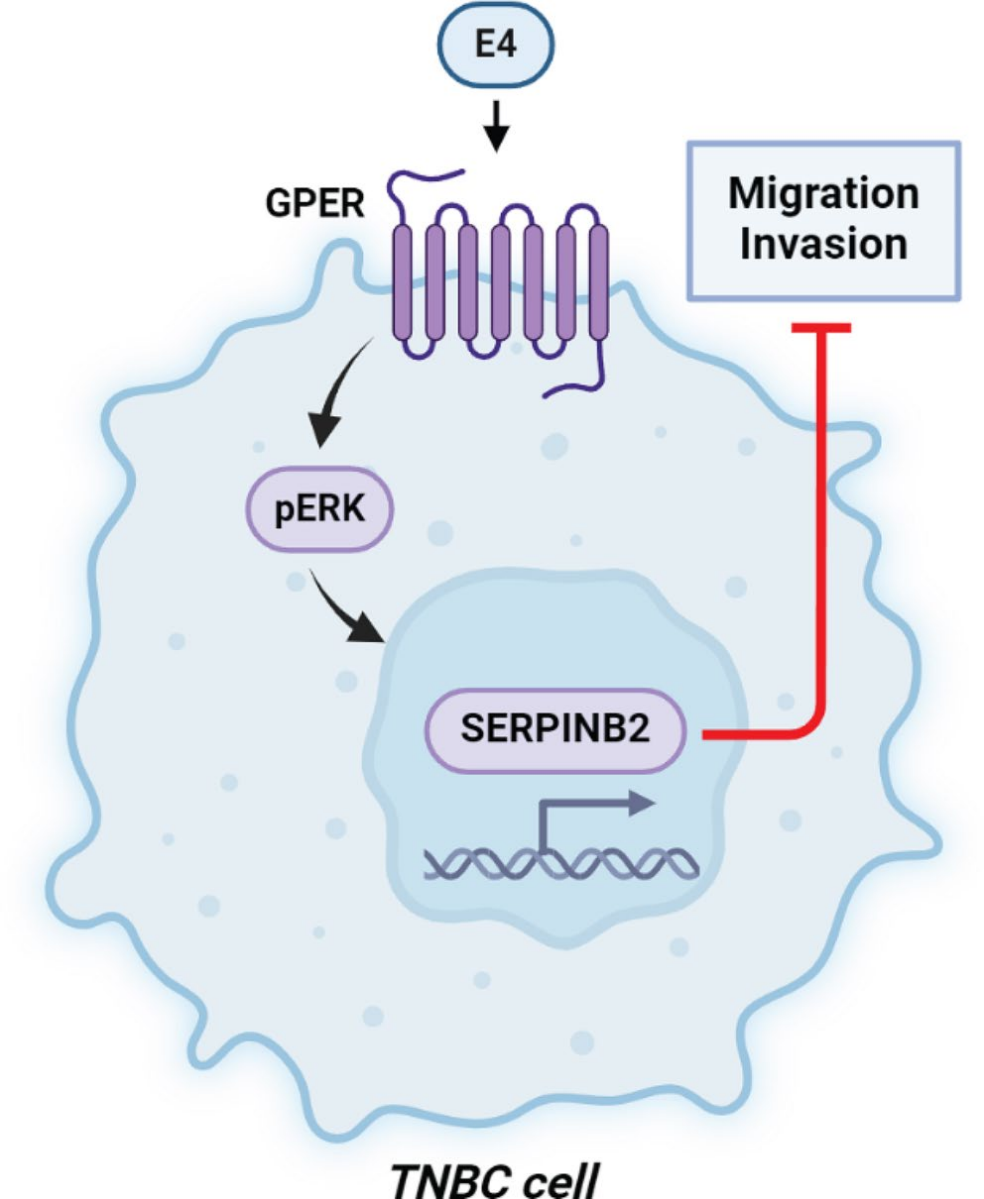
Schematic representation of the anti-migratory asnd anti-invasive effects elicited by E4 in TNBC cells via the GPER-mediated induction of SERPINB2.

Overall, the inhibitory actions triggered by E4 through the GPER/ERK/SERPINB2 pathway in TNBC cells (Fig. 7) may provide novel insights concerning the biological effects of E4 in the aggressive breast tumor subtype namely TNBC. Furthermore, our data shed new light regarding the regulation of SERPINB2 and support its tumor-suppressive action in TNBC. Nevertheless, additional studies are warranted to better define the molecular mechanisms and the biological responses to E4 in breast cancer.

## Data Availability

All data that were generated or analyzed during our study have been included in this article.

## Abbreviations

ACTB: Actin beta
DEGs: differentially expressed genes
DMEM/F-12: Dulbecco’s modified Eagle’s medium
DMFS: distant metastasis-free survival
DMSO: Dimethyl sulfoxide
DRSP: drospirenone
E1: Estrone
E2: 17β-Estradiol
E3: Estriol
E4: Estetrol
ECM: Extracellular Matrix
EGFR: Epidermal Growth Factor Receptor
ER: Estrogen receptor
FBS: Fetal bovine serum
FDA: Food and Drug Administration
FAK: focal adhesion kinase
GPER: G protein-coupled estrogen receptor
HRT: hormone replacement therapy
MD: molecular dynamics
MHT: menopausal hormone therapy
PAI-1: plasminogen activator inhibitor type-1
PAI-2: (plasminogen activator inhibitor type-2
TCGA: The Cancer Genome Atlas
TNBC: Triple-negative breast cancer
uPA: urokinase plasminogen activator
WHI: Women’s Health Initiative
